# Simplified Point-of-Care Testing for Human Pythiosis: Development of a Whole-Blood-Based Lateral Flow Immunoassay

**DOI:** 10.3390/diagnostics16050652

**Published:** 2026-02-24

**Authors:** Jidapa Szekely, Kitti Saelai, Sirida Youngchim, Siriporn Chongkae, Pornchanan Chanchay, Wiraphan Rakchang, Paramaporn Rattanaphan, Narongdet Kositpantawong

**Affiliations:** 1 Faculty of Medical Technology, Prince of Songkla University, Hat Yai 90110, Thailand; 2 Proteodiag Co., Ltd., Mueang Songkhla 90000, Thailand; pornchanan.cc@gmail.com; 3 Department of Microbiology, Faculty of Medicine, Chiang Mai University, Chiang Mai 50200, Thailand; syoungchim@gmail.com (S.Y.); siripornannt4@gmail.com (S.C.); 4 Department of Pathology, Faculty of Medicine, Ramathibodi Hospital, Mahidol University, Bangkok 10400, Thailand; ting1927@windowslive.com; 5 Department of Microbiology, Faculty of Medicine, Prince of Songkla University, Hat Yai 90110, Thailand; rattanaphan.p@hotmail.com; 6 Department of Internal Medicine, Faculty of Medicine, Prince of Songkla University, Hat Yai 90110, Thailand

**Keywords:** pythiosis, *Pythium insidiosum*, serodiagnosis, lateral flow immunoassay, LFIA, immunoblot, rapid test, antibody detection

## Abstract

**Background/Objectives**: Human pythiosis, caused by *Pythium insidiosum*, is associated with severe morbidity and high mortality when diagnosis is delayed. Culture-based diagnosis is time-consuming and may be insensitive in clinical specimens, highlighting the need for rapid point-of-care serodiagnostic tools. Here, we developed and clinically evaluated the Anti-Pin Antibody Test Strip, a whole-blood-compatible lateral flow immunoassay (LFIA) for detecting anti-*P. insidiosum* antibodies. **Methods**: Secretory protein antigens of *P. insidiosum* were prepared and conjugated to gold nanoparticles for LFIA development. Analytical performance was assessed by determining the limit of detection (LOD) using serial dilutions of pythiosis serum and by evaluating cross-reactivity with sera from patients with other infections. Interference testing examined common anticoagulants and adverse sample conditions (hemolysis, lipidemia, and icterus). Clinical performance was evaluated using 258 serum samples, comprising 48 pythiosis-positive and 210 pythiosis-negative specimens confirmed by immunoblotting and/or culture. Test results were read at 5 min. **Results**: The assay LOD was a serum titer of 1:1000. No cross-reactivity was observed across the tested infectious and immunologic panels, and no interference was detected from anticoagulants or adverse sample conditions. Whole-blood testing showed no red blood cell interference. In clinical evaluation, sensitivity was 100.00% (48/48), specificity was 95.24% (200/210), and accuracy was 96.12%, with a PPV of 82.76% and an NPV of 100.00%. **Conclusions**: The Anti-Pin Antibody Test Strip provides rapid (5 min), operationally simple serodiagnosis and may support screening/triage of suspected pythiosis, particularly where laboratory methods are limited.

## 1. Introduction

*Pythium insidiosum* is a pathogenic oomycete, a fungal-like organism phylogenetically distinct from true fungi, characterized by the production of motile biflagellate zoospores that play a central role in disease pathogenesis through direct inoculation via skin wounds or abrasions following environmental exposure [1,2]. Infection with *P. insidiosum* causes human pythiosis, a rare but life-threatening disease associated with substantial morbidity and mortality, particularly when diagnosis and treatment are delayed [3,4,5]. The organism is predominantly found in freshwater environments and, to a lesser extent, in moist soil, with a geographic distribution spanning tropical, subtropical, and temperate regions [6]. In Thailand and other endemic countries, where agriculture and outdoor water-related activities are common, frequent contact with contaminated water sources markedly increases the risk of infection [4,6,7].

Clinically, human pythiosis is classified into four major forms: vascular, ocular, cutaneous/subcutaneous, and disseminated [4]. Among these, vascular pythiosis represents the most frequently reported disseminated form, showing the most severe manifestation, followed by ocular involvement, which is often associated with poor visual outcomes [8,9]. Early and accurate diagnosis is therefore critical, as delayed recognition is strongly associated with disease progression, limb loss, and death [10]. The current diagnostic gold standard remains the isolation of *P. insidiosum* in culture followed by zoospore induction; however, this method is time-consuming, labor-intensive, and frequently insensitive when applied to clinical specimens, particularly after antimicrobial exposure [11,12]. Histopathological examination may demonstrate sparsely septate hyphae within affected tissues, but these structures are commonly misidentified as true fungal elements due to morphological similarity, leading to misdiagnosis and inappropriate antifungal therapy [12].

To address these diagnostic challenges, several serological and immunological assays have been developed, including enzyme-linked immunosorbent assays (ELISAs) and immunoblotting techniques, which have demonstrated high sensitivity and specificity for the detection of antibodies against *P. insidiosum* [13,14,15]. Despite their diagnostic accuracy, these laboratory-based assays require specialized equipment, technical expertise, and prolonged turnaround times, limiting their accessibility in routine clinical practice and resource-limited settings [13,16]. As antifungal agents are largely ineffective against *P. insidiosum*, and surgical intervention remains the cornerstone of treatment, delays in diagnosis frequently translate into poor clinical outcomes [3,12]. These limitations underscore the urgent need to develop rapid, accurate, and user-friendly diagnostic methods that can be implemented from the early stages of clinical treatment.

From a public health perspective, human pythiosis imposes a considerable burden in endemic tropical and subtropical regions, particularly among agricultural workers and individuals with occupational or recreational exposure to freshwater environments [17,18]. In Thailand alone, several hundred cases have been reported, with mortality rates exceeding 40% in vascular pythiosis despite aggressive surgical management and adjunctive therapies [4]. Increasing reports from India, Brazil, and other endemic regions further highlight pythiosis as an emerging and neglected infectious disease with serious consequences for both human and animal health [3,8,19]. Accordingly, simple, rapid, and affordable diagnostic tools can facilitate the timely initiation of appropriate treatment and may also reduce costs associated with diagnosis, therapy, and overall healthcare management.

In recent years, lateral flow immunoassays (LFIAs) have gained attention as promising point-of-care diagnostic tools for pythiosis, targeting either *P. insidiosum* antigens or host antibodies [15,16]. Although antibody-based LFIAs have demonstrated encouraging diagnostic performance, many existing platforms require serum dilution or multiple pre-analytical steps, which increase procedural complexity and the risk of operator-dependent error [12]. To overcome these limitations, we developed a novel LFIA designed to detect anti-*P. insidiosum* antibodies directly from whole blood or undiluted plasma. This platform simplifies testing procedures, shortens turnaround time, and enables application at the point of care, thereby facilitating earlier diagnosis and potentially improving clinical outcomes in patients with suspected pythiosis.

To our knowledge, no previous study has reported a whole-blood-based LFIA specifically developed for the rapid serodiagnosis of human pythiosis. The present study, therefore, aimed to evaluate the clinical performance of this novel assay and assess its potential utility as a point-of-care diagnostic tool in comparison with established reference methods.

## 2. Materials and Methods

### 2.1. Ethics Statement

The study was conducted according to international standards of Good Clinical Practice. Ethical approval was granted by the Human Research Ethics Committee (HREC), Faculty of Medicine, Prince of Songkla University, Songkhla, Thailand (approval number: REC66-481-19-2, approval date 24 December 2023). This study was conducted in accordance with the ethical principles outlined in the Declaration of Helsinki, the Belmont Report, the CIOMS Guideline, and the International Conference on Harmonization in Good Clinical Practice (ICH-GCP).

### 2.2. Protein Antigen Preparation

*Pythium insidiosum* strain CBS119452 was cultured on Sabouraud dextrose agar (SDA) for one week. Hyphal fragments (5 × 5 mm, five pieces) were then subcultured in Sabouraud dextrose broth (pH 6.9) and incubated at 35 °C with shaking at 150 rpm for 7 days. The culture was centrifuged at 4500 rpm for 15 min, and the supernatant was collected. To remove residual hyphal fragments, the supernatant was filtered through a 0.22-µm syringe filter (Sartorius, Göttingen, Germany). Secretory protein antigens were then prepared from the clarified culture supernatant using a Vivaspin® 30 centrifugal concentrator (30 kDa molecular weight cut-off; Merck, Darmstadt, Germany) according to the manufacturer’s instructions or ammonium sulfate precipitation. The protein concentration of each antigen preparation was determined prior to assay development, and protein profiles were assessed by sodium dodecyl sulfate–polyacrylamide gel electrophoresis (SDS-PAGE) under standard conditions with gel images documented for comparison. The resulting antigenic protein preparations were aliquoted and stored at −20 °C until use.

### 2.3. Development of the Anti-Pin Antibody Test Kit

#### 2.3.1. Optimization of Antigen Conjugation to Gold Nanoparticles

To optimize antigen loading for conjugation, *P. insidiosum* secretory antigens were prepared at different concentrations (25, 50, and 100 µg/mL) and conjugated to 40 nm gold nanoparticles (AuNPs). Conjugates were incorporated into lateral flow test strips and evaluated using positive-control (PC) and negative-control (NC) sera to assess signal formation and background characteristics. For pH optimization, antigen–AuNP conjugation was performed over a pH range of 6.0–8.5 using the selected antigen concentrations, and conjugation performance was assessed based on conjugate stability and strip signal behavior. Conjugate stability was monitored during preparation by visual inspection for aggregation and by the appearance/consistency of the conjugate suspension. All conjugates generated under different antigen-loading and pH conditions were subsequently tested on assembled strips using PC and NC sera.

#### 2.3.2. Evaluation of Gold Nanoparticle Conjugation Condition

**Figure 1:**
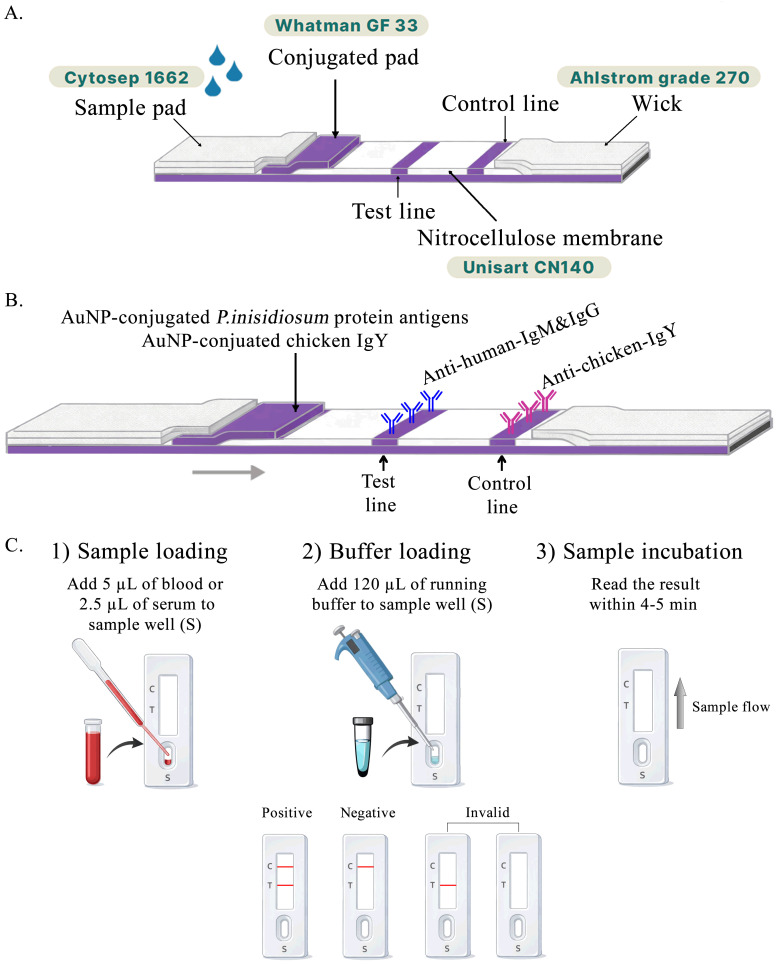
Schematic illustration of the Anti-Pin Antibody Test Strip design and test procedure. (**A**) Structural components and membrane materials used in the lateral flow strip. (**B**) Layout of key reagents on the strip. (**C**) Simplified testing workflow and interpretation.

The 40 nm colloidal gold nanoparticles (1 OD) (DCN Diagnostics, Carlsbad, CA, USA) were adjusted to the optimal pH of 6.5 for conjugation. A *P. insidiosum* protein antigen (50 µg/mL) was conjugated with 5 mL of the colloidal gold suspension. Antigen–gold conjugates were collected by centrifugation, resuspended in phosphate buffer (pH 7.4) containing 1% bovine serum albumin (BSA), and adjusted to an optical density (OD) of 20 at 450 nm. The conjugates were then applied to a glass fiber membrane (GF33; Whatman Schleicher & Schuell, Dassel, Germany) at 1 µL/mm and dried at 28 °C in a humidity control chamber to prepare the conjugate pad (Figure 1A).

#### 2.3.3. Assembly of the Anti-Pin Antibody Test Strip

A mixture of anti-human IgM (0.5 mg/mL) and anti-human IgG (0.25 mg/mL; Southern Biotech, Birmingham, AL, USA) was dispensed onto nitrocellulose membranes (Unisart CN 140; Sartorius, Göttingen, Germany) at the test line, while mouse anti-chicken IgY (0.5 mg/mL; Southern Biotech, Birmingham, AL, USA) was dispensed at the control line. The absorbent pad consisted of untreated paper (Grade 270; Ahlstrom-Munksjö, Helsinki, Finland), and the sample pad was composed of treated cellulose paper (Cytosep 1662; Cytiva, Marlborough, MA, USA, Danaher, Washington, DC, USA). All pads were assembled with a 2 mm overlap to ensure continuous capillary flow of samples and buffer along the strip (Figure 1B).

#### 2.3.4. Running Buffer Optimization

To determine the optimal running buffer for the developed lateral flow immunoassay (LFIA), phosphate buffer (20 mM, pH 7.4), Tris buffer (50 mM, pH 8.0) with 0.05% casein, and carbonate–bicarbonate buffer (CBB) (50 mM, pH 9.5) supplemented with various surfactants commonly used in immunodiagnostic applications were evaluated (Table 1). All buffers were prepared using analytical-grade reagents (Sigma-Aldrich, St. Louis, MO, USA) and filtered through 0.22 µm membrane filters prior to use to ensure sterility and the absence of particulate matter.

The performance of each buffer was evaluated based on the migration rate across the nitrocellulose membrane, test-line signal intensity, background clarity, and result reproducibility. For each condition, assays were performed in triplicate using positive controls (*P. insidiosum*-positive serum and whole blood) and negative controls (healthy serum and whole blood). Signal intensities were quantified using Image Processing and Analysis in Java (ImageJ, version 1.59g). The buffer that produced the strongest test-line signal with minimal background interference and a consistent flow rate (complete migration within 5 min) was selected as the optimal running buffer for subsequent assay development.

### 2.4. Limit of Detection (LOD)

To ensure reliable detection of low levels of anti-*P. insidiosum* antibodies, the limit of detection (LOD) of the Anti-Pin Antibody Test Strip was determined. Ten-fold serial dilutions of positive-control serum were prepared, and the analytical sensitivity was evaluated by comparing test-line signal intensities with those of the buffer-only baseline. Signal intensity was quantified using the Gel Analysis tool in ImageJ software. The LOD was defined as the lowest serum dilution that produced a visually detectable test line with a quantifiable signal intensity above background.

### 2.5. Cross-Reactivity and Interference Testing

Cross-reactivity was assessed using leftover serum samples from patients who were diagnosed with and identified as having infections positive for *Aspergillus* sp., *Candida parapsilosis*, *Cryptococcus neoformans*, *Talaromyces marneffei* and *Histoplasma capsulatum*. Additional sera from patients infected with *Acinetobacter baumannii*, *Enterobacterales*, *Staphylococcus aureus*, Streptococci, *Stenotrophomonas maltophillia*, *Orientia tsutsugamushi*, *Plasmodium vivax*, *P. falciparum*, hepatitis B virus, hepatitis C virus, human immunodeficiency virus (HIV), and severe acute respiratory syndrome coronavirus 2 (SARS-CoV-2) were also evaluated. Cross-reactivity was defined as false-positive signals at the test line. All specimens were confirmed negative for *P. insidiosum* antibody by immunoblotting (Table A1).

Interference testing was performed by spiking the anti-*P. insidiosum*-positive and anti-*P. insidiosum*-negative serum samples with commonly encountered biochemical compounds, including hemoglobin, bilirubin, and triglycerides. In addition, different types of anticoagulants used for blood collection—EDTA, heparin and sodium citrate—were evaluated. Sera with elevated rheumatoid factor, antinuclear antibody, and C-reactive protein levels were also tested. All experiments were conducted in accordance with CLSI EP07 guidelines [20]. Five microliters of the mixture were applied to the test cassette, and results were recorded after 5 min. All experiments were performed in triplicate.

### 2.6. Clinical Specimens

The study was performed between January 2024 and November 2025. A total of 258 archived plasma samples were collected from two university hospitals, Songklanagarind Hospital (Songkhla Province) and Maharaj Nakorn Chiang Mai Hospital (Chiang Mai Province). Leftover specimens were included if they were collected from patients attending healthcare facilities for suspected pythiosis and other invasive infections. No restrictions were applied regarding age or gender. 

EDTA–plasma samples were collected and transported to the laboratory under a maintained cold chain. All specimens were anonymized and relabeled with a new identification code prior to testing. Quantitative antibody levels were not available for this cohort. The same sample panel was used for immunoblotting and the developed lateral flow immunoassay (LFIA). All operators were blinded to all clinical data. Immunoblot testing and LFIA interpretation were performed independently by different operators. Before testing, samples were thoroughly mixed and equilibrated to room temperature. All results were independently interpreted by three certified medical technologists. 

Among the collected clinical samples, 48 were confirmed as pythiosis by immunoblotting, and 210 were also confirmed as anti-*P. insidiosum* antibody-negative by immunoblotting. Clinical sensitivity, specificity, positive predictive value (PPV), and negative predictive value (NPV) of the Anti-Pin Antibody Test were calculated using immunoblot analysis as the reference method.

### 2.7. Immunoblotting

Immunoblotting was performed as follows [21]. Briefly, 1 µg of secreted protein antigen from *P. insidiosum* CBS119452 was separated by SDS-PAGE and transferred to a nitrocellulose membrane. The membrane was blocked with 5% skim milk to reduce nonspecific binding. The membrane strips were incubated with individual serum samples (1:100 dilution) for 1 h at 37 °C and then washed three times with PBS containing Tween-20 (PBS-T). Bound antibodies were detected using horseradish peroxidase (HRP)-conjugated goat anti-human IgG (1:1000 dilution). Signals were developed with 4-chloro-1-naphthol as the chromogenic substrate.

### 2.8. Anti-Pin Antibody Test Strip

The prototype of the Anti-Pin Antibody Test is a qualitative chromatographic immunoassay designed to detect anti-*P. insidiosum* antibodies in human serum, plasma, or whole blood. The assay was performed as follows: 2.5–5 µL of serum/plasma or 5–10 µL of whole blood was applied to the sample well of the cassette, followed by 120 µL of running buffer. Results were interpreted within 5 min. The appearance of two colored lines, a control line (C) and a test line (T), was considered positive and indicated the presence of anti-*P. insidiosum* antibodies in the specimen. The presence of the control line alone was interpreted as a negative result (Figure 1C).

### 2.9. Data Analysis

Data were prepared and analyzed using Microsoft Excel (Microsoft Inc., Redmond, WA, USA). Confidence intervals (CIs) for sensitivity and specificity were calculated at 95%, and statistical significance was defined as a two-tailed *p* < 0.05. Statistical analyses were performed using SPSS software, version 22.0 (SPSS, Inc., Chicago, IL, USA). 

Sensitivity and specificity of the assays were calculated using a diagnostic test evaluation calculator (MedCalc Software; https://www.medcalc.org/calc/diagnostic_test.php) (Version 23.4.8; accessed on 21 December 2025), based on anti-*P. insidiosum*-positive and anti-*P.insidiosum*-negative samples confirmed by immunoblotting. Likelihood ratios (LR+ and LR−) of the assay were calculated from sensitivity and specificity estimates derived from 2 × 2 contingency tables using MedCalc Software (https://www.medcalc.org/en/calc/likelihoodratios.php) (Version 23.4.8; accessed on 3 January 2026). Ninety-five percent confidence intervals for likelihood ratios were obtained using exact methods, as implemented in MedCalc. In cases where no false-negative results were observed, LR+ approached infinity; such estimates were interpreted with caution due to limited sample size.

## 3. Results

### 3.1. Optimization of Antigen Conjugation and Running Buffer

**Figure 2:**
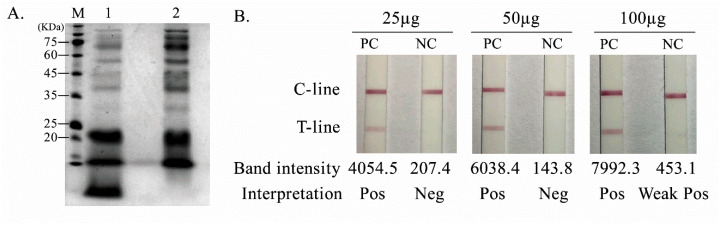
Preparation and optimization of *Pythium insidiosum* secretory protein antigen for AuNP-based lateral flow assay. (**A**) SDS-PAGE profiles of *P. insidiosum* secretory proteins concentrated using Vivaspin® 30 centrifugal concentrators (lane 1) or ammonium sulfate precipitation (lane 2). (**B**) Representative lateral flow strip results obtained using AuNP-antigen conjugates prepared with *P. insidiosum* antigen at 25, 50, and 100 µg/mL and tested with positive-control (PC) and negative-control (NC) sera. Test-line band intensities were quantified for individual strips; values > 300 were interpreted as positive (Pos).

Secretory protein antigens of *P. insidiosum* were obtained using two concentration approaches, yielding protein concentrations of 5482.0 µg/mL (Vivaspin® 30) and 2465.2 µg/mL (ammonium sulfate precipitation). SDS-PAGE demonstrated distinct protein profiles between methods (Figure 2A); ammonium sulfate precipitation produced a broad and relatively uniform band distribution across approximately 20–75 kDa, supporting its suitability for downstream antigen selection and assay development (Figure 2A).

For AuNP conjugation, antigen loading was evaluated across three conditions. An antigen concentration of 50 µg/mL generated a clear test line in positive-control sera with a clean background and no detectable signal in negative-control sera, whereas 100 µg/mL produced false positivity in negative-control sera, suggesting increased nonspecific signal at higher antigen loading (Figure 2B). Therefore, 50 µg/mL was selected for subsequent experiments.

**Figure 3:**
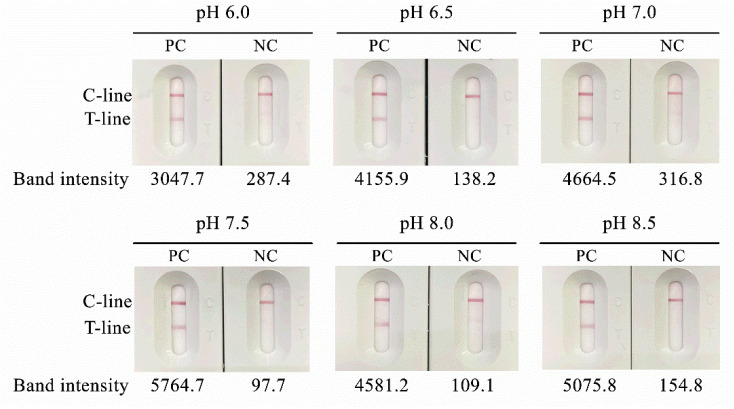
Optimization of conjugation pH for *Pythium insidiosum* antigen–AuNP conjugates. Representative lateral flow strip results obtained using AuNP conjugated with *P. insidiosum* antigen (50 µg/mL) prepared across a pH range of 6.0–8.5. Test-line band intensities were quantified for individual strips.

Conjugation pH optimization further indicated that pH 6.5 provided the strongest and most interpretable test-line signal, with stable conjugate appearance and no visible aggregation during preparation (Figure 3); thus, pH 6.5 was selected as the optimal conjugation condition.

**Figure 4:**
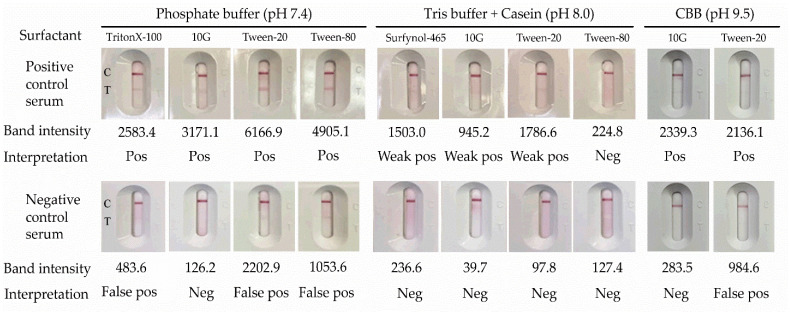
Running buffer and surfactant screening for the lateral flow assay. Representative lateral flow strip images and corresponding test-line band intensities obtained using phosphate buffer (pH 7.4), Tris-HCl buffer containing casein (pH 8.0), and carbonate–bicarbonate buffer (pH 9.5), each supplemented with different surfactants. Band intensities were quantified for individual strips; values > 300 were interpreted as positive.

Running buffer optimization showed a clear signal-to-background separation across tested formulations, with positive samples yielding T-line intensities of 483.6–6166.9 and negative samples 39.7–283.5 (Figure 4). Among the surfactants evaluated, 10G provided the most accurate classification of positive and negative specimens, whereas Tween-20 and Tween-80 were associated with false-positive signals in negative-control sera (Figure 4). Accordingly, 10G was selected for the optimized running buffer formulation.

### 3.2. Limit of Detection (LOD) 

**Figure 5:**
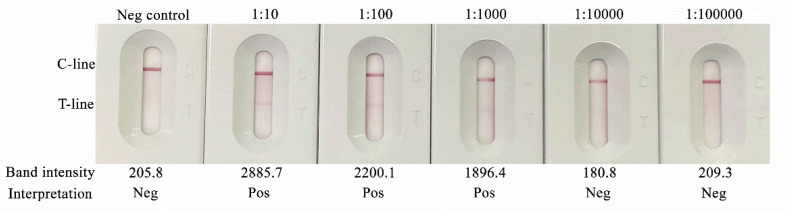
Analytical limit of detection of the Anti-Pin Antibody Test Strip using serially diluted positive-control serum. Positive-control serum was tested at dilutions from 1:10 to 1:100,000. Test-line band intensity was quantified using ImageJ; values > 300 were interpreted as positive (Pos).

The limit of detection (LOD) of the test was evaluated using 10-fold serial dilutions of positive-control serum from confirmed pythiosis patients, ranging from 1:10 to 1:100,000. Band intensity of the test line was assessed by comparison with a negative control. Accordingly, the limit of detection of the assay was determined to be at a serum titer of 1:1000 (Figure 5).

### 3.3. Cross-Reactivity and Interference Test of Anti-Pin Antibody Test Strip

**Figure 6:**
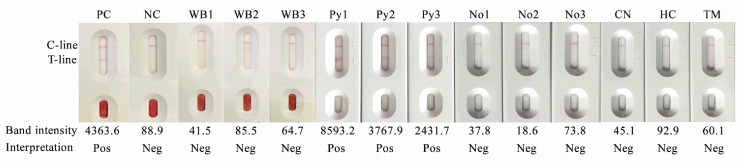
Representative results of the Anti-Pin Antibody Test Strip using whole-blood and serum samples. The figure shows lateral flow test readouts with the control line (C-line) and test line (T-line) indicated. Whole-blood testing included a positive control (PC), a negative control (NC), and whole-blood samples from healthy individuals (WB1–WB3). Undiluted serum testing included sera from patients with confirmed pythiosis (Py1–Py3), healthy individuals (No1–No3), and sera from patients with other fungal infections, including *Cryptococcus neoformans*, *Histoplasma capsulatum*, and *Talaromyces marneffei* (lanes CN, HC, and TM, respectively). Band intensity values (arbitrary units) and the corresponding interpretation (positive/negative) are shown beneath each lane. Positive results were defined by the presence of both C- and T-lines, whereas negative results showed the C-line only.

To evaluate cross-reactivity, serum samples were obtained from patients infected with *Aspergillus* spp., *Candida parapsilosis*, *Cryptococcus neoformans*, *Histoplasma capsulatum*, and *Talaromyces marneffei* (Figure 6, lanes CN, HC and TM, respectively). Additional serum samples were collected from patients with infections caused by *Acinetobacter baumannii*, *Enterobacterales*, *Staphylococcus aureus*, *Streptococcus* spp., *Stenotrophomonas maltophilia*, *Orientia tsutsugamushi*, *Plasmodium vivax*, *Plasmodium falciparum*, hepatitis B virus, hepatitis C virus, human immunodeficiency virus (HIV), and severe acute respiratory syndrome coronavirus 2 (SARS-CoV-2). Serum samples with elevated rheumatoid factors, antinuclear antibodies, and C-reactive protein were also included. All tested samples yielded negative results, indicating no detectable cross-reactivity. 

Interference studies demonstrated that commonly used anticoagulants, including heparin, EDTA, and sodium citrate, as well as serum samples exhibiting hemolysis, lipidemia, or icterus, did not interfere with the diagnostic performance of the pythiosis assay. In addition, evaluation of test performance using whole-blood and undiluted-serum samples from patients with pythiosis (Figure 6, lanes PC and Py1–3), compared with those from healthy individuals (Figure 6, lanes WB1–3 and No1–3), demonstrated that the assay yielded accurate results, with no interference from red blood cells affecting result interpretation.

### 3.4. Clinical Evaluation of the Anti-Pin Antibody Test Strip

#### 3.4.1. Pythiosis Cases and Immunoblot Analysis

A total of 258 serum samples were included in this study, comprising 48 pythiosis-positive samples and 210 pythiosis-negative samples. Pythiosis-positive cases were defined as those with positive culture results and/or positive immunoblotting for antibodies against *P. insidiosum*. Immunoblot analysis served as the reference method for antibody detection in all clinical samples.

Among pythiosis-positive patients, 48 serum samples from retrospectively confirmed cases were analyzed. The patients’ ages ranged from 10 to 77 years (mean, 43.3 years), and they were predominantly males (Supplementary Table S1). Where available, culture results were concordant with immunoblot findings, further supporting the diagnosis. Clinical specimens were obtained from diverse hospital settings, including surgical units, intensive care units, outpatient departments, and external laboratories, reflecting the heterogeneous clinical presentations and care settings of pythiosis (Supplementary Table S1).

#### 3.4.2. Clinical Performances of Anti-Pin Antibody Test Strip

The clinical performance of the Anti-Pin Antibody Test Strip was evaluated using the same cohort of 258 serum samples (Table 2). Among the 210 non-pythiosis samples, 200 were correctly identified as negative, whereas 10 samples showed false-positive results.

Test results were interpreted within 5 min following detection, based on the clear appearance of the control line. The Anti-Pin Antibody Test Strip demonstrated a sensitivity of 100.00% (48/48; 95% confidence interval [CI], 92.60 to 100.00%) and a specificity of 95.24% (200/210; 95% CI, 91.42 to 97.69%). The positive predictive value (PPV) was 82.76% (48/58; 95% CI, 72.39 to 89.78%), and the negative predictive value (NPV) was 100.00% (200/200; 95% CI, 98.17 to 100.00%). The overall diagnostic accuracy of the assay was 96.12% (95% CI, 92.99 to 98.13%) (Table 3).

In addition, likelihood ratio analysis demonstrated strong discriminative performance of the assay. A positive likelihood ratio (LR+) was infinite (95% CI: 20.73 to infinity), reflecting the absence of false-negative results in the evaluated cohort. The negative likelihood ratio (LR−) was 0.172 (95% CI: 0.098–0.303), indicating that a negative result substantially reduced the probability of pythiosis. Overall, these findings demonstrate a high level of agreement between the Anti-Pin Antibody Test Strip and immunoblot-based reference methods, supporting its reliability for the detection of pythiosis in clinical specimens.

## 4. Discussion

Human pythiosis is a severe infection in which diagnostic delay has been repeatedly linked to poor outcomes and the need for aggressive surgical management, particularly in endemic settings [3,4]. In the present study, the Anti-Pin Antibody Test Strip showed high diagnostic performance against an immunoblot-based reference standard, yielding a sensitivity of 100.00% and specificity of 95.24%, with an overall accuracy of 96.12%. The high negative predictive value (100.00%) together with a low negative likelihood ratio (LR− = 0.172) suggests that a negative result substantially decreases the likelihood of pythiosis in the evaluated cohort. Conversely, the high positive likelihood ratio indicates that a positive result provides strong diagnostic evidence, but the PPV (82.76%) remains context-dependent and should be interpreted alongside clinical features and exposure history, especially in healthcare settings with no other screening methods available [22]. Although the LR+ approached infinity, this estimate arises from zero false-negative observations rather than absolute diagnostic certainty. The wide confidence interval underscores limited precision, and such results should be interpreted cautiously, particularly in small datasets. Larger studies are required to obtain more stable estimates of likelihood ratios. Overall, performance testing indicates that the assay developed in this study may be useful as a rapid serological screening tool for suspected human pythiosis when interpreted alongside clinical findings. These results also highlight the importance of integrating test results with clinical context and performing confirmatory testing when indicated.

Serology has long served as a practical adjunct for pythiosis diagnosis because culture is time-consuming and may be insensitive in clinical specimens, while molecular testing is not consistently available in all settings [4,21,23]. Among serological methods, CFA-based ELISA has been widely reported with strong diagnostic performance and provides quantitative readouts that may support monitoring of antibody kinetics during treatment [13]. To improve standardization and reduce reliance on live-pathogen antigen preparation, peptide-based ELISAs targeting defined immunoreactive determinants (e.g., epitopes from exo-1,3-β-glucanase/PinsEXO1) have also been developed and have shown favorable performance for serodiagnosis [24].

Immunoblot/Western blot remains a commonly used confirmatory serological approach due to its high specificity and its frequent use as a reference comparator in diagnostic evaluations; however, it is labor-intensive, generally qualitative rather than quantitative, requires special equipment, and is less practical for routine testing or point-of-care use [21]. In addition, early work integrating serological and molecular assays has demonstrated that antibody recognition patterns and assay format can influence diagnostic sensitivity across clinical contexts, consistent with the view that serological performance may vary by disease form, stage, and host immune response [23].

In parallel, lateral flow immunochromatographic assays (LFIAs) have emerged as promising rapid alternatives, offering short turnaround times, minimal equipment requirements, and operational simplicity—features that are particularly relevant in endemic and resource-limited settings. Prior studies have reported favorable diagnostic performance for LFIA-based tests targeting either *P. insidiosum* antigens or host antibodies, with limits of detection in the low nanogram-per-milliliter range [15,16]. Importantly, several reports also underscore that pre-analytical complexity (e.g., serum dilution and multiple handling steps) may introduce operator-dependent variability and increase the risk of error in real-world use [15,16]. In this context, the ability of the Anti-Pin Antibody Test Strip to detect anti-*P. insidiosum* antibodies directly from whole blood or undiluted plasma represents a practical advantage that may improve workflow simplicity, reproducibility, and timely access to diagnostic information, supporting its potential utility as a rapid screening or triage tool for suspected pythiosis.

Rapid immunochromatographic (ICT) and lateral flow-type assays have been developed as user-friendly serological tools for pythiosis. The CFA-based ICT by Krajaejun et al. detected anti-*P. insidiosum* IgG with an overall sensitivity of 88% and specificity of 100% and a turnaround time of <30 min [15]. Importantly, all ocular cases were negative by both ICT and immunodiffusion, and sensitivity increased to 100% when ocular cases were excluded, underscoring the limitation of antibody-based serology in localized ocular disease and the need for cautious interpretation of negative serology in suspected ocular pythiosis [15]. This caveat has been reiterated in subsequent evaluations and reviews [4]. Comparative studies further support the utility of ICT formats. Chareonsirisuthigul et al. reported 100% sensitivity and 100% specificity for ELISA and ICT (with 100% PPV/NPV), whereas immunodiffusion was less sensitive, and hemagglutination showed poorer specificity [24]. Intaramat et al. also developed a protein A/G-based ICT applicable across human and animal hosts, showing ELISA sensitivity/specificity of 98.8%/100% versus ICT sensitivity/specificity of 90.6%/100% (accuracy 96.5%). In that study, at least one ocular serum was negative, and some weakly positive ELISA samples were ICT-negative, suggesting that rapid strip sensitivity may decrease near the lower antibody range [25]. Operationally, earlier ICT workflows may require serum dilution (e.g., 1:10,000 in the 2009 CFA-based ICT), which adds pre-analytical steps and may increase operator burden and error risk [15]. Collectively, these data support ICT/LFIA formats as practical, rapid serodiagnostic tools while highlighting known constraints in ocular disease and low-antibody states [4,15,25].

Against this backdrop, the Anti-Pin Antibody Test Strip appears to fall within the performance range reported for published ICT assays while incorporating workflow-simplifying features (rapid readout and minimal procedural steps). In our evaluation, the assay demonstrated a sensitivity of 100.00% and specificity of 95.24%, with an NPV of 100.00% and an LR− of 0.172, and results were interpretable within 5 min, supporting its potential role as a rapid screening tool when integrated with confirmatory methods as appropriate [3]. Although no false-negative results were observed, some specimens yielded weakly positive signals, which may reflect antibody levels near the assay’s effective detection threshold and/or variation in host immune response. This is consistent with prior evidence that serological sensitivity can decline in localized disease phenotypes and that some rapid ICT platforms may miss low-signal sera that remain detectable by more sensitive or quantitative methods [15,25]. Because immunoblotting is qualitative and ELISA was not performed, the relationship between band positivity and antibody titer could not be assessed directly. Future work incorporating quantitative ELISA could help determine whether these cases cluster at low antibody levels and further clarify performance across antibody-titer strata [13,24].

In endemic countries, background environmental exposure without recognized clinical disease may lead to detectable antibodies in some individuals, which can appear as “false positives” when controls are defined solely by the absence of diagnosed pythiosis. A Thai seroprevalence study demonstrated measurable seroreactivity in a subset of the general population, supporting the possibility of unrecognized exposure contributing to apparent false positivity in healthy cohorts [26]. In the present study, all immunoblot-negative/LFIA-positive discordant specimens (n = 10) were derived from healthy blood donors; however, because these were anonymized leftover samples, detailed exposure histories (e.g., freshwater contact or agricultural work) and definitive alternative diagnoses were not uniformly available, limiting further characterization beyond their immunoblot-negative status. Although nonspecific binding or low-level cross-reactive antibodies may also contribute, the lack of systematic cross-reactivity in broad panels reported in prior serological work suggests that silent exposure and population background seroreactivity are important considerations when interpreting specificity in endemic settings [16,23,26]. Accordingly, standardized clinical metadata and exposure-risk information would help clarify assay specificity and predictive values in lower-prevalence populations and enable more robust interpretation of discordant results.

Given the high NPV and low LR− observed here, the Anti-Pin Antibody Test Strip may be most useful as a rapid screening/triage tool to support earlier decision-making, particularly where immediate culture or molecular testing is limited. However, because PPV varies with prevalence and pre-test probability, positive results should be interpreted in the context of clinical phenotype and exposure history and ideally confirmed by immunoblot, culture, and/or nucleic acid-based testing when feasible [22,23,27,28]. This integrated approach aligns with the current understanding that serology provides supportive evidence, whereas direct detection methods offer definitive confirmation of active infection [4,27].

Antibody- and antigen-based LFIAs provide complementary roles for pythiosis diagnosis. The Anti-Pin Antibody Test Strip (antibody LFIA) offers a practical advantage through direct testing from whole blood/undiluted plasma with a rapid 5 min readout, supporting its use as a screening/triage tool when interpreted alongside clinical findings and confirmatory testing as needed. However, antibody detection can be less reliable in localized disease or low-antibody states, and prior studies have cautioned that negative serology should be interpreted carefully when clinical suspicion remains high [4,15,25]. In contrast, Tongchai et al. developed an antigen LFIA targeting the *P. insidiosum* antigen (PIA), providing more direct biomarker detection and potentially greater utility in earlier or selected clinical contexts; their workflow reports results within ~25 min with an analytical LOD in the low (~8) ng/mL range [16]. Overall, antibody LFIA may be best suited for rapid rule-out/triage, whereas antigen LFIA may add value for early detection, with both fitting optimally within an integrated diagnostic approach [4,16].

Several limitations warrant consideration. First, the retrospective design and the use of immunoblot as a reference standard may not fully reflect diagnostic performance in early or atypical presentations encountered in routine clinical practice. Second, the number of confirmed pythiosis-positive cases (n = 48) was necessarily limited. Although human pythiosis is increasingly recognized in Thailand, it remains an infrequent diagnosis globally, with fewer than 800 cases documented worldwide between 1980 and 2021 [8]. Moreover, among the 102 cases reported in Thailand, a notable cluster occurred toward the later part of the 1985–2003 period, underscoring the episodic nature of case accrual even in endemic settings [3]. This clinical rarity may limit statistical power and constrain the precision and generalizability of sensitivity/specificity estimates across the full disease spectrum [3,8]. Prospective validation in larger, multicenter cohorts is therefore warranted to better define real-world performance and operational robustness, including evaluation outside endemic regions where background exposure patterns and pre-test probability differ and may substantially influence predictive values and clinical interpretation [8]. Third, because immunoblot is qualitative and does not provide antibody quantitation, and ELISA was not performed in this evaluation, we were unable to explore discordant results mechanistically or to assess performance across antibody-titer strata [13,21]. Fourth, exposure histories were not available for healthy controls, which is particularly relevant in endemic settings where background seroreactivity has been documented [26]. Fifth, no cases of ocular pythiosis were included in this cohort, as serological testing is generally not required for diagnosing ocular disease. In suspected Pythium keratitis, definitive diagnosis primarily relies on testing ocular specimens (e.g., corneal scrapings) using clinical microbiology methods, including culture with zoospore induction [29] and nucleic acid-based assays. PCR has been considered the gold standard for confirmation [9,29,30]. Although ocular specimens are often limited in quantity, molecular methods remain highly suitable for diagnosing ocular pythiosis. In contrast, serology is typically uninformative because serum anti-*P. insidiosum* antibodies are usually undetectable in patients with ocular pythiosis [30]. In addition, adjunct serum biomarkers such as (1→3)-β-D-glucan and *P. insidiosum*-specific antibodies have been reported to lack utility for the diagnosis and monitoring of ocular pythiosis [4,31]. In addition, the use of culture filtrate antigens (CFAs) as capture or detector reagents may compromise assay specificity, as shared or conserved antigenic components can potentially be recognized by antibodies in environmentally exposed but clinically healthy individuals [26] and, potentially, in patients with antigenically related infections such as basidiobolomycosis [32]. This concern has been illustrated during clinical implementation of an ICT for pythiosis, where false-positive ICT results were reported in animals ultimately diagnosed with basidiobolomycosis. Mechanistic analyses suggested cross-reactivity between anti-*Basidiobolus ranarum* antibodies and multiple *P. insidiosum* proteins as a plausible explanation for apparent “false positivity”, underscoring the importance of confirmatory testing following a positive screening result [32]. However, because human basidiobolomycosis is exceedingly rare, serum specimens from affected patients were not available for inclusion in the present study.

Future studies should therefore: (i) incorporate quantitative serology (e.g., ELISA) to define assay sensitivity across antibody levels and disease stages; (ii) optimize antigen selection and purification using *P. insidiosum*-specific targets to further improve analytical specificity of the strip test; (iii) conduct prospective, multicenter validation across diverse clinical forms, including subcutaneous and ocular disease; (iv) include sites in both endemic and non-endemic regions to evaluate transportability of performance and predictive values across different pre-test probability settings; (v) evaluate real-world point-of-care workflows using whole blood; and (vi) model predictive values under varying prevalence scenarios to inform implementation algorithms and confirmatory testing strategies.

## 5. Conclusions

In summary, the Anti-Pin Antibody Test Strip demonstrated high sensitivity and specificity, with strong rule-out performance in this clinical evaluation. Its ability to detect anti-*P. insidiosum* antibodies directly from whole blood or undiluted plasma offers a practical advantage that may simplify workflow, improve reproducibility, and facilitate timely access to diagnostic information, thereby supporting its potential role as a rapid screening or triage tool for suspected pythiosis. With cautious interpretation and confirmatory testing in clinically discordant cases, this assay may contribute as a pragmatic serodiagnostic component within an integrated diagnostic strategy for pythiosis.

## Figures and Tables

**Table 1:** List of running buffers tested in this study.

Running Buffer No.	Component
Buffer 1	PBS + Triton X-100
Buffer 2	PBS + Surfactant 10G
Buffer 3	PBS + Tween 20
Buffer 4	PBS + Tween 80
Buffer 5	Tris + Casein + Surfynol 465
Buffer 6	Tris + Casein + Surfactant 10G
Buffer 7	Tris + Casein + Tween 20
Buffer 8	Tris + Casein + Tween 80
Buffer 9	Carbonate–Bicarbonate + Tween 20
Buffer 10	Carbonate–Bicarbonate + Surfactant 10G

**Table 2:** Clinical evaluation results of Anti-Pin Antibody Test Strip.

Anti-Pin Antibody Test Strip	Pythiosis Cases Diagnosed by Culture and/or Immunoblot Analysis	
	Positive	Negative
Positive	48	10
Negative	0	200
Total	48	210

**Table 3:** Sensitivity and specificity of Anti-Pin Antibody Test Strip.

Test Kit	Total (n)	% Sensitivity [95% CI]	% Specificity [95% CI]	% Accuracy [95% CI]
**Anti-Pin Antibody** **Test Strip**	258	100.00% (48/48) [92.60% to 100.00%]	95.24% (200/210) [91.42% to 97.69%]	96.12% [92.99% to 98.13%]

## Data Availability

The data may be made available upon reasonable request and are subject to approval by the ethics committee. Requests for data access should be directed to the corresponding author and will be reviewed in accordance with ethical guidelines and institutional policies.

## Supplementary Materials

The following supporting information can be downloaded at https://www.mdpi.com/article/10.3390/diagnostics16050652/s1, Table S1: Clinical data for pythiosis-positive cases confirmed by culture and/or immunoblotting compared with the Anti-Pin Antibody Test Strip results.

## Abbreviations

The following abbreviations are used in this manuscript:

CBB	Carbonate–bicarbonate buffer
CIs	Confidence intervals
EDTA	Ethylenediaminetetraacetic acid
ELISA	Enzyme-linked immunosorbent assays
HIV	Human immunodeficiency virus
LFIA	Lateral flow immunoassays
LOD	Limit of detection
LR	Likelihood ratios
NC	Negative control
NPV	Negative predictive value
OD	Optical density
PC	Positive control
PPV	Positive predictive value
SARS-CoV-2	Severe acute respiratory syndrome coronavirus 2
SDA	Sabouraud dextrose agar

## Appendix A

**Table A1:** Specimens used for analytical and clinical performance evaluations of the Anti-Pin Antibody Test Strip using immunoblot as the reference standard.

*P. insidiosum*-Positive Sample	N	Potentially Cross-Reactive Infections	N
Subcutaneous pythiosis Vascular pythiosis Disseminated pythiosis Not specified	6 36 2 4	*Fungal infection* Aspergillosis Candidiasis Cryptococcosis Histoplasmosis Talaromycosis marneffei *Bacterial infection* *Acinetobacter baumannii* *Enterobacterales* *Staphylococcus aureus* *Streptococcus* spp. *Stenotrophomonas maltophilia* *Orientia tsutsugamushi* *Parasite infection* *Plasmodium vivax* *Plasmodium falciparum* *Viral infection* Hepatitis B virus Hepatitis C virus HIV COVID-19 Dengue *Others* Rheumatoid factor-positive sample Antinuclear antibodies C-reactive protein-positive sample Healthy donor samples	1 1 3 1 2 2 4 2 1 1 2 2 1 2 2 2 4 1 2 2 2 170
