# Supplementary material for: Simplified Point-of-Care Testing for Human Pythiosis: Development of a Whole-Blood-Based Lateral Flow Immunoassay
Source: Diagnostics (Basel). 2026 Feb 24;16(5):652. doi: 10.3390/diagnostics16050652 (PMC12985033; doi:10.3390/diagnostics16050652)
Supplement: Supplementary file 1 [file diagnostics-16-00652-s001.zip › diagnostics-4168384-supplementary.pdf]

Supplementary Table S1 Clinical data for pythiosis-positive cases confirmed by culture and/or immunoblotting compared with the Anti-Pin Antibody Test Strip results

No.	Collection date	Ward	Sex	Age	Pythiosis form	Culture	Immunoblot	Anti-Pin Ab strip
1	29/05/04	N/A	N/A	N/A	Vascular	Positive	Positive	Positive
2	10/03/05	Thoracic Surgical ICU	Male	27	Vascular	ND	Positive	Positive
3	07/08/07	Thoracic Surgical ICU	Male	27	Vascular	ND	Positive	Weakly positive
4	17/12/07	Female Surgical Ward 2	Female	28	Vascular	ND	Positive	Positive
5	17/12/07	Female Surgical Ward 2	Female	28	Vascular	Positive	Positive	Positive
6	22/12/07	Thoracic Surgical ICU	Male	46	Vascular	ND	Positive	Positive
7	22/12/07	Thoracic Surgical ICU	Male	46	Vascular	ND	Positive	Weakly positive
8	27/12/07	Thoracic Surgical ICU	Male	36	Not specified	ND	Positive	Positive
9	16/01/08	Thoracic Surgical ICU	Male	36	Not specified	ND	Positive	Positive
10	29/09/08	Pediatrics Ward 3	Male	10	Vascular	Positive	Positive	Positive
11	03/10/08	Male Surgical Ward 3	Male	50	Vascular	ND	Positive	Positive
12	14/10/08	Male Surgical Ward 3	Male	50	Vascular	Positive	Positive	Positive
13	19/12/08	Male Surgical Ward 3	Male	22	Vascular	Positive	Positive	Positive
14	23/05/09	Emergency Surgical ICU	Female	61	Vascular	ND	Positive	Positive
15	25/08/09	Male Surgical Ward 3	Male	52	Vascular	ND	Positive	Positive
16	15/10/09	Male Surgical Ward 3	Male	49	Vascular	ND	Positive	Positive

17	05/03/10	General Surgery Ward	Male	48	Not specified	ND	Positive	Positive
18	07/05/10	Female Surgical Ward 2	Female	61	Vascular	ND	Positive	Positive
19	14/10/10	General Surgery Ward	Female	47	Vascular	ND	Positive	Positive
20	21/10/10	Male Orthopedics Ward 3	Male	44	Subcutaneous	ND	Positive	Positive
21	11/05/11	Ward for Buddhist monks 1	Male	54	Vascular	ND	Positive	Positive
22	16/08/12	Female Surgical Ward 3	Female	40	Subcutaneous	ND	Positive	Weakly positive
23	22/02/13	Male Surgical Ward 3	Male	39	Vascular	Positive	Positive	Positive
24	27/05/13	Male Surgical Ward 3	Male	77	Vascular	ND	Positive	Positive
25	24/04/14	General Surgery Ward	Male	41	Vascular	Positive	Positive	Positive
26	23/08/14	Female Surgical Ward 3	Female	60	Vascular	ND	Positive	Positive
27	16/09/14	Female Surgical Ward 3	Female	52	Vascular	ND	Positive	Positive
28	20/07/15	Male Surgical Ward 3	Male	50	Disseminated	ND	Positive	Positive
29	20/07/15	Male Surgical Ward 3	Male	50	Disseminated	ND	Positive	Positive
30	01/08/15	General Surgery Ward	Male	41	Vascular	ND	Positive	Positive
31	12/07/16	Emergency Surgical ICU	Male	63	Vascular	ND	Positive	Positive
32	13/07/16	Male Surgical Ward 3	Male	63	Vascular	ND	Positive	Positive
33	02/03/17	Male Surgical Ward 3	Male	N/A	Vascular	ND	Positive	Positive
34	18/09/17	Male Surgical Ward 3	Male	51	Vascular	ND	Positive	Positive
35	21/05/18	Male Surgical Ward 3	Male	51	Vascular	ND	Positive	Weakly positive

36	31/05/18	External laboratory	Female	58	Vascular	ND	Positive	Positive
37	14/11/19	General Surgery Ward	Male	62	Not specified	ND	Positive	Weakly positive
38	24/02/20	External laboratory	Female	45	Vascular	ND	Positive	Weakly positive
39	01/05/20	Female Surgical Ward 3	Female	45	Vascular	ND	Positive	Weakly positive
40	18/06/20	External laboratory	Male	32	Vascular	ND	Positive	Positive
41	03/12/20	General Surgery Ward	Male	50	Vascular	ND	Positive	Positive
42	4/02/2021	Male Surgical Ward	Male	55	Vascular	Positive	Positive	Positive
43	06/02/23	OPD 22	Female	55	Vascular	ND	Positive	Positive
44	17/02/23	OPD 22	Male	33	Subcutaneous	ND	Positive	Positive
45	26/07/23	OPD 22	Female	26	Subcutaneous	ND	Positive	Positive
46	30/07/24	Male Surgical Ward 1	Male	45	Subcutaneous	ND	Positive	Positive
47	01/08/24	Male Surgical Ward 1	Male	45	Subcutaneous	ND	Positive	Positive
48	12/09/24	Male Surgical Ward 3	Male	25	Vascular	ND	Positive	Positive
